# Molecular Mechanisms Underlying Antimicrobial Resistance in Mycobacteria

**DOI:** 10.3390/ijms27156893

**Published:** 2026-08-01

**Authors:** Paula López-Roa, Jaime Esteban, María-Carmen Muñoz-Egea

**Affiliations:** 1 Department of Clinical Microbiology, Hospital Universitario 12 de Octubre, 28041 Madrid, Spain; 2 Department of Clinical Microbiology, IIS-Fundación Jiménez Díaz, Universidad Autónoma de Madrid (UAM), 28040 Madrid, Spain; 3 CIBERINFEC-CIBER de Enfermedades Infecciosas, 28029 Madrid, Spain

**Keywords:** mycobacteria, nontuberculous mycobacteria, *Mycobacterium tuberculosis*, antimicrobial resistance, molecular mechanisms

## Abstract

Antimicrobial resistance in mycobacteria arises from a complex interplay of intrinsic and acquired mechanisms that collectively limit the efficacy of current therapeutic options. Intrinsic resistance is largely driven by the low permeability of the mycobacterial cell envelope, the activity of efflux pumps, and the presence of drug-modifying enzymes, which together restrict intracellular drug accumulation and contribute to broad baseline tolerance. This review integrates resistance mechanisms of both *M. tuberculosis* and *M. abscessus*, two clinically relevant mycobacteria that share core molecular pathways while exhibiting species-specific determinants that complicate treatment. Additional intrinsic factors, including biofilm formation and stress-induced adaptive responses, further enhance persistence and reduce susceptibility to multiple drug classes. Acquired resistance predominantly results from chromosomal mutations affecting drug targets or prodrug activation pathways, such as *katG*, *inhA*, *rpoB*, *gyrA*, and *pncA* in *Mycobacterium tuberculosis*, leading to high rates of multidrug-resistant and extensively drug-resistant disease. In nontuberculous mycobacteria, species-specific determinants—including inducible macrolide resistance mediated by *erm(41)* in *M. abscessus*, plasmid-mediated *erm (55)* variants, *rrl* and *rrs* mutations, diverse enzymatic inactivation systems, and regulatory alterations in the MarR family that result in inducible resistance to drugs such as ethionamide —generate highly variable resistance profiles that complicate treatment. Recent advances in molecular diagnostics, including PCR-based assays, whole-genome sequencing, CRISPR-based diagnostic platforms, AI-assisted diagnostics, and emerging multi-omics approaches, have improved the detection of resistance-associated mutations and enhanced understanding of mycobacterial pathophysiology. In parallel, new therapeutic agents and optimized regimens offer promising avenues to overcome resistance, although emerging resistance to novel drugs underscores the need for continued surveillance. This review synthesizes current knowledge on the molecular basis of resistance in *M. tuberculosis* and NTM, highlighting implications for diagnosis, treatment, and future research.

## 1. Background and Scope of the Review

*Mycobacterium tuberculosis* remains one of the most significant human pathogens and is responsible for tuberculosis (TB), which continues to be the leading cause of death from a single infectious agent worldwide, with a substantial burden of both latent and active disease [1]. Latent TB infection constitutes a long-term reservoir for future disease, with progression to active TB determined by host immune status and time since initial infection [1]. The clinical importance of *M. tuberculosis* is further enhanced by its capacity to evade host immunity, persist in a dormant state, and cause a wide spectrum of disease [2].

In parallel, nontuberculous mycobacteria (NTM) have emerged as important opportunistic pathogens. These environmental organisms are increasingly recognized as causes of pulmonary and extrapulmonary infections, particularly in patients with preexisting lung conditions or compromised immunity. The most common etiologic agents of NTM pulmonary disease include *Mycobacterium avium* complex, *M. kansasii*, and *M. abscessus*, and their incidence is rising globally [3,4]. In addition, a history of pulmonary TB may predispose individuals to NTM infection due to residual structural damage, while NTM does not increase the risk of TB but does complicate the differential diagnosis in high-incidence areas. The prevalence of NTM lung disease is increasing globally, with rates ranging from 1 to 6 cases per 100,000 people and annual increases of 8–10% in several countries [3,4].

Antimicrobial resistance represents a major global health threat in mycobacterial infections. In 2022, the WHO updated the classification and previous definitions of TB drug resistance [5]. These changes better reflect current treatment practices. According to the latest definitions, Multidrug-resistant TB (MDR-TB) is resistant to at least isoniazid and rifampicin, pre-extensively drug-resistant TB (pre-XDR-TB) refers to multidrug-resistant or rifampicin-resistant TB (MDR/RR-TB) with additional resistance to any fluoroquinolone, and extensively drug-resistant TB (XDR-TB) has also been redefined and now refers to MDR/RR-TB with resistance to any fluoroquinolone plus at least one additional Group A drug (such as bedaquiline or linezolid). XDR-TB is associated with higher mortality, prolonged and poorly tolerated treatment regimens, and lower cure rates (MDR-TB ~50%, XDR-TB ~30%) [5,6,7]. At the same time, NTMs also exhibit distinct patterns of acquired resistance. In MAC, resistance to macrolides is primarily associated with mutations in *rrl* and the presence of *erm* genes, whereas in *M. abscessus*, *erm(41)*-mediated inducible resistance coexists with acquired resistance due to mutations in *rrl* and *rrs*, resulting in strains with clinically relevant resistance to macrolides and aminoglycosides [3,4]. Inadequate or incomplete treatment regimens, poor adherence, and delayed diagnosis of drug-resistant TB rapidly select *M. tuberculosis* strains with genetically acquired resistance [8]. Although newer agents such as bedaquiline have improved outcomes, emerging resistance to these drugs has already been reported [9,10]. In NTM infections, the development of drug resistance is also facilitated by prolonged treatment courses, suboptimal drug penetration in structurally damaged lungs, intrinsic species-specific tolerance, and delayed diagnosis, which often leads to extended exposure to ineffective regimens. These factors collectively promote selection of resistant subpopulations and mirror the clinical drivers of resistance observed in *M. tuberculosis* [3,4].

Similarly, NTM exhibit both intrinsic and acquired resistance mechanisms, particularly among rapidly growing species such as *M. abscessus*, which pose significant therapeutic challenges and often require individualized, susceptibility-guided treatment regimens due to their broad spectrum of resistance determinants and variable drug response profiles [4]. Overall, the rise of MDR/XDR-TB and resistant NTM underscores the urgent need for rapid diagnostics, optimized treatment regimens, and continuous surveillance to improve patient outcomes.

This review provides a comprehensive overview of the most important molecular mechanisms that underlie antimicrobial resistance in *M. tuberculosis* and NTM. It first addresses the biological bases of intrinsic resistance, encompassing the cell envelope’s low permeability; biofilm-associated tolerance; the role of drug-modifying enzymes such as β-lactamases, acetyltransferases, and methyltransferases; and stress adaptation responses. It further explores the molecular mechanisms underlying acquired resistance, highlighting critical mutations in drug target genes, efflux pump–mediated drug extrusion, and enzymatic inactivation, with emphasis on species-specific determinants such as *erm(41)* in *M. abscessus*. Finally, the review summarizes recent advances in diagnostic and therapeutic strategies, encompassing molecular techniques for resistance detection, such as PCR and sequencing-based assays, and the development of new drugs and advanced treatment regimens designed to enhance efficacy and address emerging resistance.

In addition to summarizing established resistance mechanisms, this review integrates recent conceptual advances—including multi-omics biomarkers, CRISPR-based functional genomics, AI-assisted diagnostics, host-directed therapies, and innovative nanocarrier strategies—that have emerged in the last few years and were not covered in earlier reviews. These elements provide an updated and forward-looking perspective on the molecular basis of resistance in *M. tuberculosis* and NTM.

## 2. Biological Basis of Intrinsic Resistance

**Figure 1:**
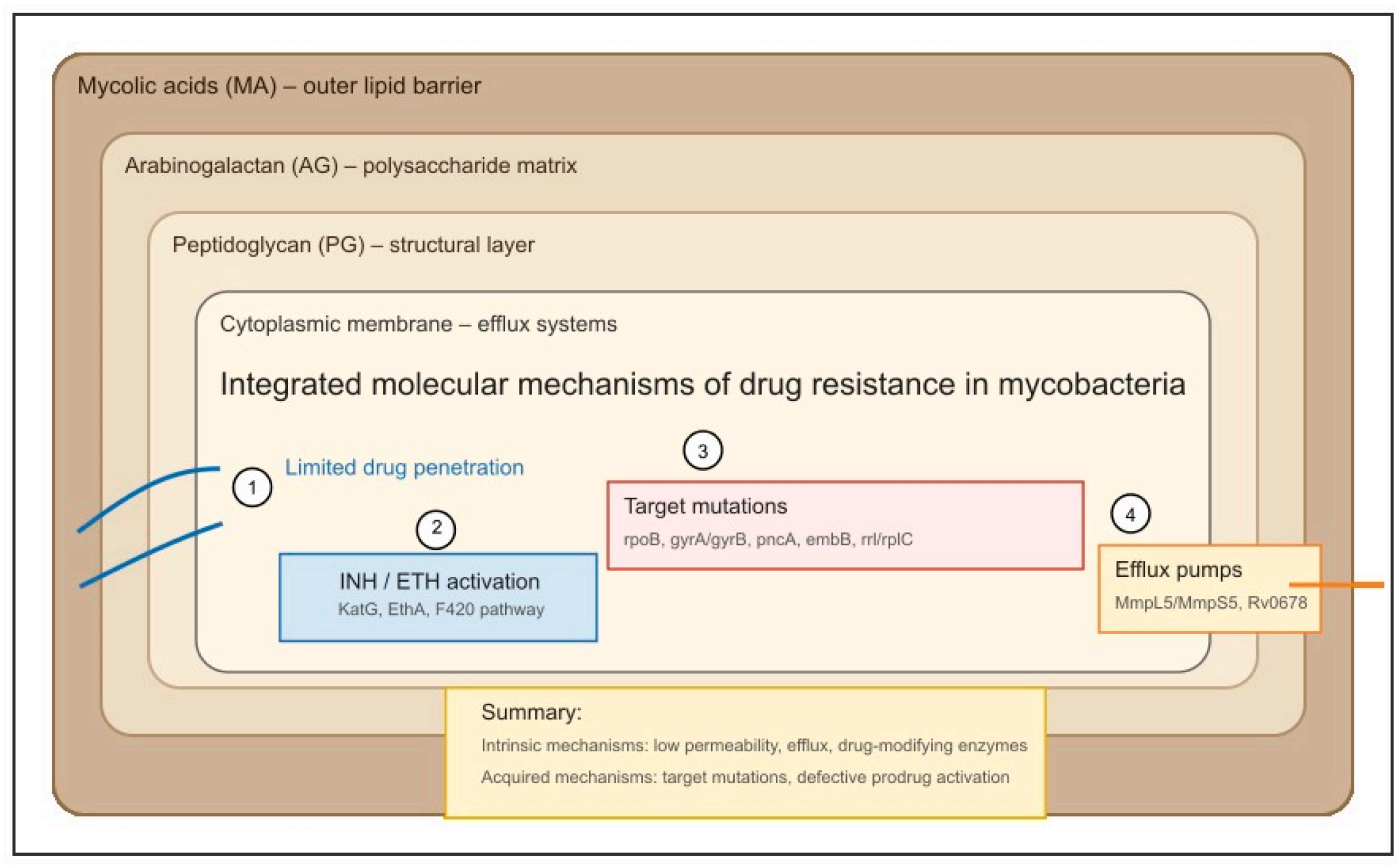
Integrated molecular mechanisms of drug resistance in mycobacteria. This original figure summarizes the key intrinsic and acquired mechanisms contributing to antimicrobial resistance in *Mycobacterium tuberculosis* and nontuberculous mycobacteria. Created with Inkscape 1.3.2.

The underlying mechanisms of antimicrobial resistance in mycobacteria are multifactorial and include both intrinsic and acquired processes (Figure 1).

The principal mechanisms of intrinsic antimicrobial resistance in mycobacteria are low permeability of the cell envelope, drug-modifying and drug-target-modifying enzymes, efflux pumps and intrinsic resistance genes. These mechanisms act collectively to confer broad intrinsic resistance to many antibiotic classes, necessitating the use of specialized antimycobacterial agents for effective treatment. These intrinsic mechanisms are mediated by well-defined genetic determinants, including the inhA and kasA pathways involved in mycolic-acid biosynthesis [11,12,13,14,15], the blaC β-lactamase responsible for broad β-lactam hydrolysis [16], rRNA methyltransferases such as ermMT that confer intrinsic macrolide resistance [11], and constitutive efflux systems such as LfrA and MmpL5/MmpS5 that reduce intracellular drug accumulation [12,17,18].

### 2.1. Low Permeability of the Mycobacterial Cell Envelope

In reference to the low permeability of the mycobacterial cell envelope, it is notable that these organisms possess a unique, thick, lipid-rich cell envelope. The mycobacterial cell envelope follows a four-layer asymmetric architecture composed of an outer capsule, an outer lipid leaflet enriched in trehalose dimycolate and PDIMs, the mAGP core (mycolic-acids–arabinogalactan–peptidoglycan), and the plasma membrane. This multilayered envelope acts as a barrier to the entry of numerous antibiotics, and its low permeability is a key determinant of intrinsic resistance that significantly limits the effectiveness of most conventional antibiotic therapies [11,12,13,14,15].

Inhibition of mycolic acid synthesis represents a major vulnerability of the mycobacterial cell envelope and is the target of several key antimycobacterial drugs. Mycolic acids are essential long-chain fatty acids forming the mycolyl–arabinogalactan–peptidoglycan complex, and their biosynthesis depends on the FAS-I and FAS-II systems, particularly the enoyl-ACP reductase InhA. Isoniazid and ethionamide inhibit this pathway after activation by KatG or EthA, forming adducts that block InhA. Resistance arises mainly through *katG* S315T mutations or *inhA* promoter overexpression. Newer nitroimidazoles (delamanid, pretomanid) also inhibit methoxy- and keto-mycolic acid synthesis via F420-dependent activation, with resistance linked to *ddn*, *fgd1*, and *fbiA–D* mutations [19,20,21,22]. Beyond *M. tuberculosis*, several envelope-associated genes are conserved across NTM species and similarly contribute to low permeability. Rapidly growing mycobacteria such as *M. abscessus* possess homologues of *kasA*, *kasB*, *inhA*, and FAS-II components involved in mycolic acid biosynthesis, as well as species-specific outer-membrane lipids (e.g., GPLs) that further reduce drug penetration [3,4]. In MAC and *M. abscessus*, envelope-associated efflux systems (e.g., *MmpL*, *MmpS* transporters) also participate in intrinsic tolerance, indicating that cell-envelope-mediated impermeability is a shared resistance determinant across both MTBC and NTM.

### 2.2. Drug-Modifying and Target-Modifying Enzymes

On the other hand, mycobacteria express a variety of enzymes that can inactivate or modify antibiotics before they reach their targets. Examples include β-lactamases, particularly BlaC in *M. tuberculosis*, which hydrolyze the β-lactam ring of penicillins and cephalosporins, rendering them inactive and explaining the limited efficacy of β-lactams unless combined with potent inhibitors. Aminoglycoside-modifying enzymes such as the acetyltransferase Eis and the phosphotransferase Aph catalyze acetylation or phosphorylation of drugs like kanamycin, amikacin, and streptomycin, reducing their affinity for the 30S ribosomal subunit and contributing to intrinsic or low-level resistance. Additional enzyme families—including monooxygenases, oxidoreductases, and ADP-ribosyltransferases—further expand the spectrum of antibiotic inactivation across nontuberculous species. A distinct and clinically relevant category is rRNA methyltransferases, which modify specific nucleotides within 23S or 16S rRNA and thereby prevent antibiotic binding. In the *M. tuberculosis* complex (MTBC), *ermMT* methylates the macrolide-binding site, conferring intrinsic macrolide resistance and precluding the use of clarithromycin or azithromycin for TB treatment [11,16].

In NTM, particularly rapidly growing species such as *M. abscessus*, *M. bolletii*, *M. massiliense*, *M. chelonae*, and *M. fortuitum*, the repertoire of drug-modifying enzymes is broader and clinically more relevant. These organisms encode multiple β-lactamases (e.g., Mabβ, CβlA) with extended hydrolytic activity, diverse aminoglycoside-modifying enzymes including ADP-ribosyltransferases, and inducible rRNA methyltransferases such as *erm(41)* and *erm(55)*, which mediate inducible macrolide resistance and frequently lead to treatment failure. This expanded enzymatic arsenal constitutes a major pillar of intrinsic resistance in NTM [11,16,23,24].

### 2.3. Efflux Pump Systems

It is also important to note that mycobacteria encode multiple efflux pump systems that actively export a variety of antibiotics out of the cell, reducing intracellular drug concentrations and contributing to intrinsic resistance. Constitutive efflux—mediated by transporters such as LfrA or MmpL5/MmpS5—operates at baseline levels and contributes to high MICs for drugs including fluoroquinolones and aminoglycosides. Stress-inducible efflux systems (e.g., EfpA, Tap) are transiently upregulated upon antibiotic exposure, providing an adaptive response that enhances short-term survival. In addition, mutations that upregulate efflux pump expression, particularly in regulatory genes such as Rv0678, generate acquired resistance by driving persistent overexpression and increased drug extrusion [12,16,17,18].

### 2.4. Biofilm-Associated Intrinsic Resistance

Intrinsic resistance in mycobacterial biofilms describes the elevated drug tolerance observed when mycobacteria grow as multicellular communities encased in an extracellular matrix. Biofilm formation leads to several protective adaptations: the matrix physically impedes antibiotic diffusion, the microenvironment within biofilms fosters slow growth and metabolic dormancy, and there is an enrichment of persister cells with heightened stress tolerance [25,26,27,28].

Although biofilm formation is best characterized under experimental conditions, structured biofilm-like aggregates have been described in human TB lesions (caseous necrosis and cavitary disease) and in MAC pulmonary disease, where they correlate with increased drug tolerance and chronic infection. These observations support the clinical relevance of biofilm-associated persistence [29].

In *M. tuberculosis* and NTM, biofilm-associated cells are significantly less susceptible to antibiotics compared to planktonic cells, often necessitating physical disruption of the biofilm or adjunctive therapies to achieve effective treatment [26,27]. Examples include reduced inhibitory and bactericidal activity of isoniazid and ethambutol in biofilm-associated cells, whereas rifampicin and clofazimine retain higher efficacy under biofilm conditions. Biofilm-associated genetic determinants such as *mmpL*, *pstC* and *sigE* in *M. tuberculosis*, and GPL biosynthesis genes and *whiB7* in NTM, further contribute to biofilm formation and drug tolerance [30,31].

## 3. Molecular Mechanisms of Acquired Resistance in Mycobacteria

Intrinsic resistance mechanisms have been summarized in Section 2; here we focus exclusively on acquired resistance, which arises from mutations affecting drug targets, activation pathways, and regulatory networks.

Building on earlier reviews such as Miotto et al. [32], which systematically summarized the full spectrum of resistance mutations in *M. tuberculosis* in 2017, this section integrates post-2020 evidence, including newly validated resistance determinants such as *rv2983* (also designated *fbiD*), updated World Health Organization mutation catalogues, and mechanisms associated with recently introduced drugs.

Acquired resistance in mycobacteria arises primarily through chromosomal mutations, transcriptional regulation, and enzymatic mechanisms, which together reduce the effectiveness of antimicrobial agents. Major contributors to resistance encompass mutations in drug target genes (e.g., *katG*, *rpoB*, *embB*, *gyrA*) that impair drug binding or activation, inducible efflux systems (e.g., MmpL, Rv1258c) that lower intracellular antibiotic concentrations, and enzymatic mechanisms, including species-specific factors such as *erm(41)* in *M. abscessus* that inactivate or modify antimicrobials [33,34]. With a few exceptions—such as plasmid-mediated erm genes in *M. abscessus* and mobile genetic elements in environmental mycobacteria—mycobacterial resistance is predominantly acquired through chromosomal mutations rather than horizontal gene transfer. Microevolution during infection promotes heteroresistance and the presence of diverse resistant subpopulations, posing significant challenges to both treatment and infection control [35,36,37,38].

### 3.1. Acquired Resistance in Mycobacterium tuberculosis

Genetic mutations in drug target genes represent the primary mechanism of acquired resistance in *M. tuberculosis*, affecting both first-line and critical second-line therapies, as described in the World Health Organization Catalogue of mutations in *MTBC* and their association with drug resistance (2023), which systematically compiles relevant variants and classifies them according to the strength of evidence linking each mutation to drug resistance [39].

#### 3.1.1. Isoniazid

Isoniazid resistance is predominantly mediated by mutations in *katG* and the *inhA* promoter. The S315T substitution in *katG* is the most prevalent chromosomal mutation worldwide, conferring high-level resistance by impairing the catalase–peroxidase activity required for prodrug activation and thereby abolishing drug efficacy [40]. Mutations in the *inhA* promoter, particularly C-15T, contribute to low-level isoniazid resistance by upregulating expression of the target enoyl-acyl carrier protein reductase and are associated with cross-resistance to ethionamide [41]. Co-occurrence of these mutations in the same strain can lead to enhanced resistance levels, emphasizing their critical contribution to the worldwide prevalence of isoniazid-resistant *M. tuberculosis*.

#### 3.1.2. Rifampicin

Similarly to the mechanisms underlying isoniazid resistance, high-level rifampicin resistance in *M. tuberculosis* is predominantly mediated by chromosomal mutations within the 81-base-pair rifampicin-resistance-determining region (RRDR) of the *rpoB* gene. The S531L substitution is the most prevalent globally, disrupting the RNA polymerase β-subunit binding interface and preventing effective drug interaction while largely preserving enzyme function in the absence of the drug [42]. Other RRDR mutations, including H526Y/D and D516V, are less frequent but may also confer resistance, with variable effects on rifampicin and rifabutin susceptibility [43].

The most clinically relevant resistance-conferring mutations in *M. tuberculosis*, affecting both first-line and second-line drugs, are summarized in Table 1, together with their molecular mechanisms and diagnostic implications.

#### 3.1.3. Pyrazinamide

Pyrazinamide (PZA) is a prodrug converted by pyrazinamidase into pyrazinoic acid, which disrupts membrane energetics and is particularly active under acidic conditions. Resistance to PZA is primarily mediated by mutations in the *pncA* gene, which encodes pyrazinamidase, the enzyme responsible for converting PZA into its active metabolite, pyrazinoic acid. These mutations are highly heterogeneous and distributed across the coding sequence and promoter region, resulting in partial or complete loss of enzymatic activity and failure to activate the drug [44]. Additional mechanisms include mutations in *rpsA*, encoding ribosomal protein S1, which can confer resistance in isolates lacking *pncA* alterations [45], and mutations in *panD*, encoding aspartate decarboxylase, representing an alternative resistance pathway in a minority of strains [46]. Low-level resistance can also result from overexpression of efflux pumps, including *Rv0191, Rv3756c, Rv3008*, and *Rv1667c*, which increase pyrazinoic acid export, although this mechanism is less common than *pncA*-mediated resistance [59]. In addition, *Mycobacterium bovis* exhibits intrinsic resistance to pyrazinamide due to a non-functional pyrazinamidase, resulting in natural drug inactivation failure [60].

Additional recently characterized determinants include *pepQ* (associated with clofazimine/bedaquiline cross-resistance) and efflux-related genes such as *Rv0191*, *Rv3756c*, and *Rv3008*, which contribute to drug tolerance and may precede fixed mutations [61,62,63,64]. Detection of these genes improves the results of genotypic drug susceptibility testing by capturing low-level resistance variants associated with tolerance.

#### 3.1.4. Ethambutol

Mutations in the *embB* gene represent the principal mechanism of ethambutol resistance in *M. tuberculosis*, altering arabinosyl transferase activity, disrupting cell envelope biosynthesis, and diminishing drug susceptibility. The most frequent substitutions occur at codon 306 (Met306Ile/Val/Leu), while additional mutations at codons 406 and 497, within the *embB*300–500 region, and in the *embC–embA* intergenic region also contribute to resistance. These *embB* mutations are present in the majority of ethambutol-resistant isolates and show a strong correlation with phenotypic resistance, although they may occasionally be detected in susceptible strains and can be associated with broader multidrug-resistant profiles [40,47,48].

#### 3.1.5. Fluoroquinolones

Beyond first-line agents, resistance to fluoroquinolones, key second-line drugs, is predominantly mediated by amino acid substitutions in *gyrA* codons 88–94, which disrupt the quinolone-binding pocket of DNA gyrase A, reduce drug affinity, and elevate minimum inhibitory concentrations (MICs) for ofloxacin, levofloxacin, and moxifloxacin. The most clinically relevant substitutions are A90V, S91P, and D94G/A/N/Y, with D94G being the most prevalent globally; codon 94 mutations generally confer higher-level resistance than A90V or D94A. Structural and functional studies confirm that these alterations directly impair drug–enzyme interactions, and the majority of fluoroquinolone-resistant isolates harbor mutations within this region, allowing sensitive detection via molecular diagnostics [49,50,65,66]. Mutations in *gyrB*, although less frequent, can further elevate MICs and broaden cross-resistance, particularly when co-occurring with *gyrA* mutations, underscoring the importance of screening both genes for accurate resistance profiling in MDR and XDR-TB [50,51,67,68]. Mutations at codon 94 (especially D94G) consistently produce higher MICs than A90V or D94A, reflecting their stronger disruption of the quinolone-binding pocket.

#### 3.1.6. Aminoglycosides

Resistance to aminoglycosides, particularly kanamycin and amikacin, is primarily mediated by mutations in the 16S rRNA gene (*rrs*) and the promoter region of *eis*, which upregulates aminoglycoside-modifying enzymes. These genetic alterations reduce drug binding to the ribosome, conferring variable levels of resistance. Mutations in *rrs* are generally associated with high-level resistance, whereas *eis* promoter mutations often confer low- to moderate-level resistance, particularly to kanamycin.

#### 3.1.7. Bedaquiline and Clofazimine

Following a similar molecular paradigm, resistance to newly introduced anti-tuberculosis agents is primarily mediated by chromosomal mutations disrupting target binding or activation pathways. Bedaquiline resistance is predominantly mediated by mutations in the Rv0678 gene, which derepresses the MmpS5/MmpL5 efflux pump, reducing intracellular drug concentrations and conferring cross-resistance to clofazimine [52,53]. Less frequently, mutations in *atpE* (the drug target ATP synthase) and *pepQ* contribute to resistance [69]. *Rv0678* mutations often emerge following prior exposure to bedaquiline or clofazimine and may coincide with additional resistance to fluoroquinolones or injectable agents, negatively affecting treatment outcomes and carrying potential for transmission [54].

#### 3.1.8. Nitroimidazoles

Nitroimidazole resistance (delamanid and pretomanid) is driven by mutations in genes involved in prodrug activation and F420 biosynthesis, including *ddn, fgd1*, and *fbiA–D*, which impair enzymatic conversion and drug activity [55,56,70]. Additional mechanisms include mutations in *rv2983*, a guanylyltransferase required for F420 biosynthesis, and specific *ddn* or *fbiA* variants that may selectively affect susceptibility to one agent over the other [56,71,72]. As noted in recent studies, *rv2983* corresponds to *fbiD*, and both designations refer to the same gene involved in F420 biosynthesis [56].

#### 3.1.9. Linezolid

Linezolid resistance similarly arises via mutations in target genes that alter the binding site on the 50S ribosomal subunit. Key determinants include mutations in the 23S rRNA gene (*rrl*), notably G2814T and G2270T, and in the ribosomal protein L3 gene (*rplC*), particularly T460C, which results in the C154R substitution and elevated minimum inhibitory concentrations [57,58,73]. The *rplC* T460C mutation (C154R) produces a structural distortion of ribosomal protein L3 that markedly reduces linezolid binding, explaining its strong phenotypic impact.

### 3.2. Resistance in Non-Tuberculous Mycobacteria

The molecular mechanisms of acquired resistance in NTM are primarily driven by chromosomal mutations, enzymatic modification, transcriptional regulation, and, more recently, plasmid-mediated gene acquisition. This section reviews these major categories of acquired resistance mechanisms in NTM, while also highlighting representative species-specific resistance profiles where relevant. Chromosomal point mutations in drug target genes are a major determinant, with substitutions in the 23S rRNA gene (*rrl*) conferring high-level macrolide resistance through alteration of the drug-binding site, most commonly at positions A2058 and A2059, while mutations in the 16S rRNA gene (*rrs*) mediate aminoglycoside resistance. Alterations in other targets, such as *gyrA* for fluoroquinolones, have also been reported [16,24].

In MAC, macrolide resistance is primarily mediated by *rrl* mutations and inducible *erm* genes, whereas *M. kansasii* exhibits predictable rifampicin susceptibility but variable fluoroquinolone resistance. In *M. abscessus* and related rapidly growing species, resistance can further arise through inducible or acquired expression of antibiotic-modifying enzymes, including erythromycin ribosome methyltransferases (*erm* genes, e.g., *erm(41)*), aminoglycoside acetyltransferases, phosphotransferases, β-lactamases, and rifamycin ADP-ribosyltransferases. These enzymes chemically modify or inactivate antibiotics, producing resistance that develops during or following drug exposure [24,74]. Alterations in transcriptional regulatory genes, particularly within the *MarR* family of transcriptional repressors, have been implicated in the derepression of downstream resistance determinants, thereby facilitating drug-specific adaptive resistance [75,76], which are discussed in detail in the dedicated subsection below. In addition, emerging evidence indicates that plasmid-mediated resistance contributes to genetic adaptability in NTM, with plasmid-borne genes such as *erm(55)* associated with transposons and secretion systems that may facilitate horizontal gene transfer and accelerate dissemination of resistance determinants [23,77]. These mechanisms differ fundamentally from intrinsic resistance, mediated by low cell envelope permeability and constitutive efflux pump activity, and are typically selected under antibiotic pressure, often resulting in treatment failure and severely restricted therapeutic options [16,24,74]. Intrinsic resistance arises from low permeability and constitutive efflux, whereas acquired resistance results from target mutations, inducible methyltransferases, and plasmid-borne determinants.

#### 3.2.1. Plasmid-Mediated Macrolide Resistance via *erm(55)*

The discovery of *erm(55)* represents the first documented case of plasmid-mediated macrolide resistance in mycobacteria. This gene encodes a novel 23S rRNA methyltransferase that is phylogenetically distinct from previously described RGM erm genes, sharing less than 65% amino acid identity with known homologs. Three variants have been identified: the plasmid-borne *erm(55)P*, the transposon-associated *erm(55)T*, and the chromosomal *erm(55)C*. Screening studies detected *erm(55)* in 3.8% of *M. chelonae* isolates, while genomic analyses identified *erm(55)P* in *M. iranicum* and *M. obuense* isolates dating back to 2008, suggesting that plasmid-mediated macrolide resistance has circulated undetected among RGM for more than a decade [78].

Functional characterization of the three *erm(55)* variants confirmed their capacity to mediate macrolide resistance, providing definitive evidence for the role of *erm(55)* as a resistance determinant [79].

The epidemiological importance of *erm(55)* is highlighted by the identification of 30 related plasmids from clinical isolates collected between 1997 and 2024 across at least six RGM species (*M. chelonae*, *M. bacteremicum*, *M. grossiae*, *M. iranicum*, *M. neoaurum*, and *M. obuense*). These plasmids exhibit a conserved backbone and frequently encode heavy-metal resistance operons, indicating that environmental co-selection likely contributes to the sustained maintenance of macrolide resistance in the absence of direct antimicrobial exposure. Together, these findings indicate long-term horizontal dissemination of conjugative *erm(55)*-carrying plasmids among RGM species [23,77].

The prototype plasmid *pMchErm55* encodes both type IV (T4SS) and type VII (T7SS) secretion systems, which cooperate in mycobacterial plasmid conjugation. Although direct conjugative transfer of *pMchErm55* has not yet been demonstrated experimentally, its genetic architecture strongly supports transfer competence [80,81].

More broadly, pan-genomic analyses of the *M. abscessus complex* indicate that most resistance-associated proteins are located within the accessory genome and are linked to mobile genetic elements, including plasmids and prophages. These observations suggest that *erm(55)* may represent only the first recognized example of a larger reservoir of plasmid-mediated antimicrobial resistance determinants in NTM [23].

#### 3.2.2. MarR-Family Regulators and Adaptive Resistance in *M. abscessus*

Increasing evidence indicates that MarR-family transcriptional regulators play a central role in the adaptive resistance networks of *M. abscessus*. These proteins typically act as transcriptional repressors, and their inactivation through mutations or conformational changes leads to derepression of downstream genes involved in antimicrobial resistance and stress adaptation [82].

The contribution of MarR-dependent regulation to ethionamide (ETH) resistance was demonstrated through transposon sequencing and genomic analyses of spontaneous resistant mutants. Loss-of-function mutations in a MarR-family regulator resulted in constitutive expression of downstream targets and selective resistance to ETH, without affecting susceptibility to amikacin or clarithromycin. These findings underscore the specificity of resistance pathways in *M. abscessus* and are particularly significant given the potent in vitro activity of ETH [76].

Subsequent studies have expanded the role of MarR-like regulators beyond ETH resistance. Mutations in MAB2648c have been associated with reduced nitroxoline susceptibility through deregulation of MmpS5-MmpL5 efflux pumps, whereas alterations in the TetR-family regulator MAB2885 confer resistance to tedizolid and linezolid by upregulating the MAB2302-MAB2303 efflux system. Functional complementation, transcriptomic analyses, and DNA-binding assays confirmed the direct regulatory role of these transcription factors in controlling efflux-mediated resistance [82,83].

Transcriptomic studies further demonstrate that antibiotic exposure triggers both drug-specific and shared adaptive responses. Ribosome-targeting agents, including clarithromycin, amikacin, and tigecycline, induce a common transcriptional program characterized by activation of ribosomal genes and the whiB7 regulon, while simultaneously reducing susceptibility to multiple classes of translation inhibitors, thereby promoting transient cross-resistance phenotypes [75].

ETH resistance is further reinforced by intrinsic mechanisms. The NADH pyrophosphatase NudC (MAB3513c) inactivates the ETH-NAD adduct, limiting drug activity before inhibition of mycolic acid synthesis can occur. Genetic disruption of nudC significantly increases ETH susceptibility, and structural studies have identified unique features of the enzyme that may facilitate future inhibitor development. Together, MarR-mediated transcriptional derepression and NudC-dependent drug inactivation illustrate the multilayered nature of ETH resistance in *M. abscessus*, combining acquired regulatory mutations with intrinsic protective mechanisms [84,85].

At a broader evolutionary scale, analysis of 5,617 clinical isolates revealed positive selection across multiple resistance-associated genes, indicating ongoing evolutionary pressure on antimicrobial resistance determinants in *M. abscessus*. Together with the emergence of mobile elements such as *erm(55)*-carrying plasmids, these findings highlight the remarkable genomic plasticity of RGM and support integrated surveillance strategies combining phenotypic and molecular approaches [86].

Table 2 provides an overview of the major genetic determinants of antimicrobial resistance in NTM, highlighting species-specific mechanisms and their relevance for clinical management and molecular diagnostics.

## 4. Diagnostic and Therapeutic Advances

Recent advances in the management of mycobacteria, including both *M. tuberculosis* and NTM, encompass both diagnostics and therapies.

Early detection of emerging drug resistance can be improved by monitoring high-confidence genetic biomarkers, including *katG* S315T and *inhA* promoter mutations for isoniazid [41,89], *rpoB* RRDR substitutions for rifampicin [90], *pncA* loss-of-function variants for pyrazinamide [91], *gyrA* codon 90–94 mutations for fluoroquinolones [92], *rrs* A1401G for aminoglycosides [93], and *Rv0678* mutations associated with early bedaquiline and clofazimine resistance [94,95]. Transcriptional indicators such as efflux pump overexpression (e.g., *Rv1258c*, *MmpL5/MmpS5*) and *eis* promoter activation can also precede fixed mutations and signal emerging resistance [96,97].

Diagnostic platforms were condensed into a mechanism-based framework directly linked to Section 2 and Section 3. Xpert MTB/RIF Ultra and Xpert MTB/XDR detect the core resistance mutations described earlier—*rpoB* (rifampicin), *katG/inhA* (isoniazid), *gyrA/gyrB* (fluoroquinolones), and *rrs/eis* (aminoglycosides)—providing rapid identification of the chromosomal determinants summarized in Table 1. NAAT-based line probe assays similarly target these canonical loci, but remain limited to hotspot regions and may miss variants outside the RRDR, *pncA, embB* or *Rv0678*, which are increasingly relevant in clinical practice. Whole-genome sequencing expands detection to emerging determinants such as *Rv0678, pepQ, ddn, fgd1* and *fbiA–D*, directly corresponding to the novel resistance mechanisms detailed in Section 3.1.7, Section 3.1.8 and Section 3.1.9. In contrast, metagenomic NGS provides broad genomic coverage but shows blind spots for plasmid-mediated resistance (e.g., *erm(55)* in *M. abscessus*) and transcriptional regulatory mutations (MarR-family), which are central to inducible resistance in NTM (Section 3.2). This integrated mechanism–technology approach replaces previous enumerative descriptions and aligns diagnostic capabilities with the molecular basis of resistance.

AI-assisted design has been proposed as a promising strategy to optimize lipidic and polymeric nanocarriers for antimycobacterial therapy. Although current systems have been developed through conventional formulation approaches, existing evidence shows that nanoparticle-based delivery platforms can enhance intracellular drug accumulation and improve therapeutic efficacy in respiratory infections [98]. In parallel, advances in AI for nanomaterial engineering suggest that machine-learning-guided optimization could accelerate the development of next-generation nanocarriers with improved targeting and performance [99]. Another potential use of AI for tuberculosis diagnosis is image analysis, which has been described for the analysis of acid-fast stains [100] and chest X-rays [101] with excellent negative predictive value, which means that negative results can be considered true negatives, while positive results need to be reviewed by expert technicians. However, these results are likely to improve with the use of new AI tools capable of learning from mistakes through machine learning and deep learning, which will lead to improved diagnosis of these diseases. AI-based resistance prediction remains constrained by training-set bias, limited representation of NTM species, and the absence of curated mutation catalogues comparable to those available for *M. tuberculosis* [102].

Matrix-assisted laser-desorption-ionization–time-of-flight mass spectrometry (MALDI-TOF MS) has emerged as a valuable tool for rapid, cost-effective species-level identification of NTM from cultured isolates, substantially shortening turnaround times and enhancing laboratory efficiency. Recent refinements in sample preparation protocols and continuous expansion of spectral reference databases have improved the discriminatory capacity of MALDI-TOF MS, particularly for closely related or newly described mycobacterial species, although performance remains dependent on database completeness and culture quality [103,104,105].

Whole-genome sequencing (WGS) now forms the foundational layer of molecular diagnostics in mycobacteriology, providing high-resolution data for phylogenetic reconstruction, outbreak investigation, transmission mapping, and prediction of antimicrobial resistance profiles. Building on this genomic baseline, integrated multi-omics approaches—including transcriptomics, proteomics, lipidomics, and metabolomics—are being explored to identify novel microbial and host-derived biomarkers and to refine early diagnostic strategies. Complementary artificial intelligence (AI) and machine learning frameworks aim to integrate these heterogeneous molecular, microbiological, radiological, and clinical datasets to improve diagnostic accuracy and clinical decision-making. Despite their promise, all three layers face shared limitations, including variable standardization across platforms, incomplete validation for NTM species, and the need for robust clinical datasets to ensure reproducibility and real-world applicability [103].

Despite these substantial advances, important limitations continue to challenge the implementation and clinical impact of modern mycobacterial diagnostics. Molecular assays may show reduced sensitivity in paucibacillary specimens, extrapulmonary tuberculosis, or pediatric populations, and they are unable to discriminate between viable and non-viable bacilli, limiting their value for treatment monitoring and assessment of microbiological cure [3,4]. Furthermore, many commercial NAATs target a restricted set of predefined resistance-conferring mutations, potentially overlooking rare, lineage-specific, or novel resistance mechanisms, particularly in NTM species with complex resistance profiles [36].

Although MALDI-TOF MS has significantly accelerated NTM identification, it generally requires prior culture growth, which remains time-consuming for slow-growing mycobacteria, and its accuracy may be compromised for uncommon species or mixed infections. WGS and multi-omics platforms, while highly informative, remain constrained by high costs, long turnaround times in routine settings, substantial bioinformatic demands, and the need for specialized infrastructure and expertise, limiting their widespread adoption, particularly in low- and middle-income countries. Additionally, the clinical validation, standardization, regulatory approval, and ethical oversight of AI-driven diagnostic tools are still evolving, posing barriers to their integration into routine diagnostic algorithms. Addressing these limitations through technological innovation, harmonized standards, and equitable implementation strategies will be essential to fully realize the potential of advanced diagnostics in improving the global management of mycobacterial diseases.

Therapeutically, the introduction of new and repurposed drugs has transformed the treatment landscape. Therapeutic advances focus on improving efficacy and tolerability.

Short-course all-oral regimens such as BPaLM/BPaL represent a major therapeutic advance because they directly target the molecular pathways described in this review. Their efficacy depends on the absence of resistance to bedaquiline (*Rv0678*, *atpE*), pretomanid (F420-dependent activation via *ddn*, *fgd1*, *fbiA–D*), linezolid (*rrl*, *rplC*), and moxifloxacin (*gyrA/gyrB*). These regimens illustrate how modern treatment strategies increasingly rely on precise molecular characterization of resistance determinants to guide individualized therapy.

Regarding NTM, for pulmonary disease caused by *Mycobacterium avium complex* (MAC), the recommended treatment continues to be a combination of a macrolide (either azithromycin or clarithromycin), ethambutol, and rifampicin, administered for a minimum of 12 months after cultures become negative, in accordance with guidelines from the American Thoracic Society, the Infectious Diseases Society of America, and the European Respiratory Society [33,106].

Inhaled liposomal amikacin is approved for refractory *Mycobacterium avium* complex (MAC) pulmonary disease, and ongoing trials are evaluating the use of bedaquiline, linezolid, and novel agents [107,108,109].

For pulmonary disease caused by *M. abscessus*, treatment remains particularly challenging due to intrinsic and inducible macrolide resistance mediated by the *erm(41)* gene. Current regimens typically include a multidrug combination of macrolides (when active); intravenous agents such as amikacin, imipenem or cefoxitin; and oral agents including clofazimine or linezolid, although outcomes remain suboptimal and prolonged therapy is often required [4].

In contrast, *M. kansasii* pulmonary disease is generally more susceptible to standard antimycobacterial therapy, with recommended regimens including rifampicin, ethambutol, and either isoniazid or a macrolide, typically administered for at least 12 months after culture conversion, achieving high treatment success rates [4].

Adjunctive strategies under investigation include host-directed therapies, immunotherapies, and bacteriophage therapy, particularly for drug-resistant and refractory cases [109,110,111]. The integration of molecular diagnostics with individualized therapy is a key focus for future management [103].

These diagnostic advances are particularly relevant because they enable detection of resistance-associated variants before phenotypic expression, improving early therapeutic decision-making. In parallel, emerging host-directed therapies and AI-assisted nanocarrier design offer complementary strategies to enhance drug delivery and mitigate resistance development.

### CRISPR-Based Tools

Recent advances in genome-editing technologies, particularly CRISPR-based systems, have provided powerful tools to dissect the genetic basis of drug resistance in mycobacteria.

CRISPR-based technologies have transformed the study of drug resistance in mycobacteria by enabling precise genetic manipulation, high-throughput functional genomics, and targeted gene silencing. CRISPR interference (CRISPRi), based on catalytically inactive dCas9, allows tunable repression of essential genes, facilitating the identification of vulnerabilities involved in cell envelope synthesis, efflux, and redox metabolism. This approach has been particularly valuable for dissecting resistance mechanisms linked to *katG, inhA, ddn*, and F420-dependent pathways. CRISPRi libraries have also enabled genome-wide chemical–genetic profiling to predict drug synergies and resistance liabilities [112,113]. CRISPR-based base editing and CRISPR-Cas systems are currently being explored in *M. tuberculosis* but remain in an early developmental stage. These platforms are well established in *M. smegmatis* and are only being gradually optimized for *M. tuberculosis*, whose low homologous recombination efficiency and slow growth rate hinder precise gene editing. CRISPR-based base editing and CRISPRi platforms are used to dissect drug-resistance mechanisms through intracellular genetic manipulation. In contrast, CRISPR-Cas12 and Cas14 detection systems function as cell-free diagnostic tools for highly sensitive identification of drug-resistant strains [114,115]. The CRISPR-Cas12 systems, and the more recently developed Cas14a platform, have been applied for the detection of resistance in *M. tuberculosis*. An example of this is the direct detection in sputum of rifampicin- and isoniazid-resistant *M. tuberculosis* by identifying mutations in *rpoB*, *katG*, and *inhA*, with high sensitivity and specificity [116].

Given that IS6110 is widely recognized as a molecular marker in the diagnosis of tuberculosis, a recently presented study used a cascade strategy combining DNA enzymes with the CRISPR-Cas12a system to accurately identify the IS6110 sequence of *M. tuberculosis*. This approach exhibits high specificity and sensitivity, differentiating it from non-tuberculous mycobacteria and other respiratory pathogens [117].

## 5. Discussion

Antimicrobial resistance in mycobacteria represents one of the most pressing challenges in modern infectious disease medicine [1,2].

Genotype–phenotype discordance remains a major challenge in mycobacterial drug resistance. Mutations in *embB, pncA* and *gyrB* frequently show variable MICs across strains and species, complicating interpretation of molecular results and limiting the predictive value of genotypic assays [44,47,50].

The fitness cost associated with resistance mutations is highly heterogeneous. Variants such as *katG S315T* and *rpoB S531L* preserve enzymatic function and favour transmission, whereas several *gyrA D94* substitutions impose substantial metabolic burden, reducing competitive fitness in the absence of drug pressure [40,42,49].

Heteroresistance and within-host microevolution generate mixed bacterial populations during treatment. These dynamic subpopulations complicate molecular detection, contribute to discordant phenotypic results, and may facilitate stepwise acquisition of multidrug resistance [35,36,37,38].

A central question is why drug resistance in the MTBC arises almost exclusively through chromosomal mutations, whereas plasmid-mediated resistance is increasingly recognized in NTM, particularly RGM [74,75,76]. This divergence reflects fundamental ecological and evolutionary differences: MTBC is an obligate human pathogen with limited opportunities for horizontal gene transfer, a clonal population structure, and strong purifying selection, all of which favor mutation-driven adaptation [32,39]. In contrast, NTMs inhabit diverse environmental niches rich in mobile genetic elements, conjugative plasmids, and phage-derived cargo genes, creating a genomic landscape that facilitates acquisition and dissemination of plasmid-encoded resistance determinants such as *erm(55).* These contrasting evolutionary contexts shape not only how resistance emerges but also how rapidly it spreads across species [74].

These observations indicate that resistance evolution in mycobacteria cannot be interpreted through a single mechanistic framework. MTBC and NTM follow distinct evolutionary logics shaped by ecological niche, genome architecture, and horizontal-gene-transfer potential [32,74]. Recognizing these differences is essential for designing species-tailored diagnostic algorithms and treatment strategies.

Future work should integrate WGS-based mutation catalogues, plasmid surveillance, accessory-genome analysis, and AI-driven predictive models to capture both mutation-driven and plasmid-mediated resistance trajectories [39,56]. This integrative approach will be essential for improving diagnostic accuracy, anticipating emerging resistance reservoirs, and guiding the development of next-generation therapeutics.

In conclusion, understanding the molecular basis of antimicrobial resistance in mycobacteria is essential for guiding both diagnostic and therapeutic strategies [1,2]. Continued research into the genetic, biochemical, and regulatory mechanisms of resistance will be critical for the development of new drugs, the optimization of treatment regimens, and the design of effective public health interventions [5,6,7].

By integrating post-2020 molecular insights, CRISPR-based functional genomics, multi-omics biomarkers, and AI-driven therapeutic innovations, this review provides an updated synthesis that reflects the rapidly evolving landscape of mycobacterial resistance [17,18,19,20,56].

## 6. Conclusions

A comprehensive understanding of the molecular mechanisms underlying antimicrobial resistance in MTBC and NTM is essential for improving diagnostic accuracy, guiding individualized therapy, and anticipating emerging resistance. Continued integration of genomic, functional, and AI-driven approaches will be critical for developing next-generation diagnostic and therapeutic strategies.

## Figures and Tables

**Table 1:** Major genetic determinants of antimicrobial resistance in *Mycobacterium tuberculosis.*

Drug	Gene(s)	Gene Function	Key Mutations/Regions	Molecular Mechanism of Resistance	Clinical and Diagnostic Relevance	Reference
Isoniazid (INH)	*katG*	Catalase-peroxidase (INH activation)	S315T (most prevalent), S315N	Loss of catalase-peroxidase activity; failure of prodrug activation	High-level INH resistance; routinely detected by LPAs and WGS	[40]
	*inhA promoter*	Enoyl-ACP reductase	C-15T, A-16G	Overexpression of InhA target enzyme; low-level resistance and ethionamide cross-resistance	Important for regimen adjustment	[41]
Rifampicin (RIF)	*rpoB*	RNA polymerase β subunit	S531L, H526Y/D, D516V (RRDR)	Reduced binding of rifampicin to RNA polymerase β-subunit	Surrogate marker of MDR-TB; cornerstone of rapid diagnostics	[42,43]
Pyrazinamide (PZA)	*pncA*	Pyrazinamidase	Highly heterogeneous mutations (coding/promoter)	Loss of pyrazinamidase activity; lack of drug activation	Challenging molecular detection; best assessed by WGS	[44]
	*rpsA, panD*	Ribosomal protein S1/Aspartate decarboxylase	Rare substitutions	Impaired drug–target interaction	Adjunctive resistance mechanisms	[45,46]
Ethambutol (EMB)	*embB*	Arabinosyl transferase	M306I/V/L; mutations at codons 406, 497	Altered arabinosyl transferase; defective cell envelope synthesis inhibition	Moderate diagnostic value; may appear in susceptible strains	[47,48]
Fluoroquinolones	*gyrA*	DNA gyrase A	A90V, S91P, D94G/A/N/Y	Reduced binding to DNA gyrase A	Core determinant of XDR-TB	[49]
	*gyrB*	DNA gyrase B	N538D, E540V	Additional impairment of DNA gyrase function	Increases resistance level when combined with gyrA	[50]
Aminoglycosides (amikacin, kanamycin)	*rrs*	16S rRNA	A1401G, C1402T	Altered 16S rRNA; reduced ribosomal binding	High-level injectable resistance	[16,24]
	*eis promoter*	Acetyltransferase	−10G→A, −14C→T	Overexpression of acetyltransferase	Low- to moderate-level resistance	[16,24]
Bedaquiline	*Rv0678*	Regulator of MmpS5/MmpL5 efflux	Frameshift, nonsense, missense mutations	Derepression of MmpS5–MmpL5 efflux pump	Cross-resistance; increasingly monitored by WGS	[51,52]
	*atpE*	ATP synthase subunit c	A63P (rare)	Reduced drug binding to ATP synthase	Rare but high-impact mutations	[53]
	*pepQ*	Peptidase	Frameshift, missense mutations	Efflux-linked tolerance	Low-level resistance marker	[53]
Clofazimine	*Rv0678*	Efflux regulator	Frameshift, missense	Derepression of efflux pump	Cross-resistance	[51,52]
Nitroimidazoles (delamanid, pretomanid)	*ddn, fgd1, rv2983 (fbiD)*	F420-dependent prodrug activation	Loss-of-function mutations	Impaired F420-dependent prodrug activation	Emerging relevance in short-course regimens	[54,55,56]
Linezolid	*rrl (23S rRNA)*	23S rRNA	G2814T, G2270T	Reduced binding to 50S ribosomal subunit	Increasingly reported in XDR-TB	[56,57,58]
	*rplC*	Ribosomal protein L3	T460C (C154R)	Structural alteration of ribosomal protein L3	Important WGS target	[56,57,58]
	*clpC1*	Clp protease	N-terminal domain	Altered *clp* protease activity	Important WGS target; documented association with PZA resistance	[56,57,58]

**Table 2:** Major genetic determinants of antimicrobial resistance in non-tuberculous mycobacteria (NTM).

Drug	Species (Examples)	Gene(s)	Gene Function	Key Mutations/Mechanisms	Clinical and Diagnostic Relevance	Reference
Macrolides	*M. abscessus*, MAC	*rrl* (23S rRNA)	Target of macrolides; mutations confer high-level macrolide resistance	A2058, A2059	High-level macrolide resistance; critical for therapy selection	[4,87]
	*M. abscessus*	*erm(41)*	Inducible methyltransferase causing macrolide resistance in *M. abscessus* subsp. *Abscessus*	Functional vs. truncated variants	Inducible macrolide resistance; must be assessed before treatment	[88]
Aminoglycosides	*M. abscessus*, MAC	*rrs* (16S rRNA)	Aminoglycoside binding site; mutations confer resistance	A1408G (species-dependent)	High-level resistance to amikacin	[88]
Fluoroquinolones	MAC, *M. kansasii*	*gyrA*	DNA gyrase; mutations confer fluoroquinolone resistance	Variable substitutions	Reduced DNA gyrase binding; variable clinical impact	[17,18]
β-lactams	*M. abscessus*	*blaMab*	β-lactamase; hydrolyzes β-lactams	High basal expression; structural variants	Intrinsic and acquired β-lactam resistance	[16,88]
Rifamycins	*M. abscessus*	*arr*	ADP-ribosyltransferase; inactivates rifamycins	ADP-ribosyltransferase activity	Inactivation of rifamycins	[16]
Tetracyclines	*M. abscessus* *M. chelonae* *M. fortuitum*	*tet(M)* efflux systems	Ribosomal protection protein; tetracycline resistance Active efflux of multiple antibiotics	Ribosomal protection/efflux	Emerging resistance	[12,17,18,33]
Ethionamide	*M. abscessus*	*MarR*-like regulators	Transcriptional repressors controlling efflux and stress responses	Derepression of resistance genes	Adaptive resistance under drug pressure	[76,83]
Multidrug resistance	*M. abscessus* *M. chelonae* *M. fortuitum*	Efflux pumps (*MmpL*, *ABC*)/*erm(55)*	Lipid transporters and ABC pumps mediating multidrug efflux Inducible macrolide resistance in *M. chelonae*	Overexpression	Reduced intracellular drug accumulation	[12,17,23,78,79]
Plasmid-mediated resistance	*M. avium* *M. intracellulare* *M. kansasii*	transposon-associated genes	Mobile elements carrying resistance determinants	Horizontal gene transfer	Emerging epidemiological concern	[35,77]

## Data Availability

No new data were created or analyzed in this study. Data sharing is not applicable to this article.

## Abbreviations

The following abbreviations are used in this manuscript:

AI	Artificial Intelligence
ATP	Adenosine Triphosphate
ATS	American Thoracic Society
BPaL	Bedaquiline Pretomanid Linezolid
BPaLM	Bedaquiline Pretomanid Linezolid Moxifloxacin
CRISPRi	CRISPR interference
EMB	Ethambutol
ERS	European Respiratory Society
ETH	Ethionamide
HIV	Human Immunodeficiency Virus
INH	Isoniazid
LPA	Line Probe Assay
MAC	Mycobacterium avium Complex
MALDI-TOF MS	Matrix-Assisted Laser Desorption Ionization–Time of Flight Mass Spectrometry
MDR	Multidrug Resistant
MDR-TB	Multidrug-Resistant Tuberculosis
MIC	Minimum Inhibitory Concentration
mNGS	metagenomic Next-Generation Sequencing
MTBC	Mycobacterium tuberculosis Complex
NAAT	Nucleic Acid Amplification Test
NGS	Next-Generation Sequencing
NTM	Nontuberculous Mycobacteria
PCR	Polymerase Chain Reaction
RGM	Rapidly growing mycobacteria
PZA	Pyrazinamide
RIF	Rifampicin
RR	Rifampicin Resistant
RRDR	Rifampicin Resistance-Determining Region
TB	Tuberculosis
WHO	World Health Organization
WGS	Whole-Genome Sequencing
XDR	Extensively Drug Resistant
XDR-TB	Extensively Drug-Resistant Tuberculosis
